# Prophylactic Effects of Ivermectin and Closantel Treatment in the Control of *Oestrus ovis* Infestation in Sheep

**DOI:** 10.3389/fvets.2021.798942

**Published:** 2022-01-18

**Authors:** Hornblenda Joaquina Silva Bello, José Gabriel Gonçalves Lins, Ana Cláudia Alexandre de Albuquerque, Gabriel Badial Ferreira, Mônica Regina Vendrame Amarante, Alessandro Francisco Talamini do Amarante

**Affiliations:** ^1^School of Veterinary Medicine and Animal Science, UNESP - São Paulo State University, Botucatu, Brazil; ^2^Institute of Biociences, UNESP - São Paulo State University, Botucatu, Brazil

**Keywords:** myiasis, Ovis aires, lambs, bot fly, Oestridae

## Abstract

The sheep nasal bots *Oestrus ovis* is parasite of the nasal cavities and sinuses of small ruminants causing oestrosis, one of the most frequent parasitic diseases in sheep and goats. The widely use of ivermectin and closantel by the sheep breeders in the treatment and prophylaxis of gastrointestinal nematodes resulted in widespread cases of anthelmintic resistance. However, there is no report about cases of *O. ovis* with drug-resistance. In this study, we evaluated the prophylactics and therapeutic effects of both antiparasitics in sheep with *O. ovis* natural infestation. The trial was carried out from early December 2019 to March 2020, with 30 crossbred males lambs allocated into three groups of 10 animals each: control (without treatment), treated with ivermectin (0.2 mg/kg subcutaneously) and treated with closantel (10 mg/kg orally). The animals were kept together grazing the same pasture area. The treatment groups were drenched in two occasions 70 days apart: on 5th December 2019 and on 13th February 2020. On 19th March 2020, all lambs were slaughtered. The lamb heads were removed and sectioned along their longitudinal and sagittal axis to search for larvae. Recovered *O. ovis* larvae were counted and identified according to their developmental stage (L1, L2, and L3). Seven of the control lambs were infested with *O. ovis* larvae ranging from six to 17 larvae (11.6 mean infestation intensity). All recovered larvae from control group were intact and active. Three animals treated with ivermectin had *O. ovis* larvae (1–3 larvae), however they were dead and in degeneration. The animals treated with closantel did not have any larvae. The clinical suggestive signs of oestrosis were scarce over the experimental period. The averages of daily weight gain were similar (*p* > 0.05) among groups. Closantel and ivermectin had high efficacy against oestrosis and *O. ovis* parasitism did not hinder the performance of lambs.

## Introduction

*Oestrus ovis* L. (Diptera: Oestridae) is a cosmopolitan parasite with a higher prevalence in tropical regions. It causes cavitary myiasis in small ruminants, once the larvae are an obligate parasite of the nasal and sinus cavities of sheep and goats (1).

In endemic areas, the *O. ovis* larvae have been found in humans' eyes and nasopharyngeal airway. Cases of ocular affections are likely to occur more frequently than currently notified, because many cases without complications are unreported (2).

The female fly flies around the head of its host to deposits larvae at a few centimeters from sheep nostrils (3). In case of the fly presence, the animals hide the muzzle on the soil or in the others sheep's wool, swing head and sneezing, which leads to distress. The larvae hooks and spine irritate the nasal mucosal, provoking inflammation and mucous nasal discharge. At the same time, this irritating action secures the production of an inflammatory exudate, which count for larva's feeding (4). Besides frequent sneezes, sheep infested with *O. ovis* larvae may also present difficulty in breathing, hyporexia, and weight loss. In heavy infestation, animals may have related secondary bacterial infection in the lungs (5).

In a trial conducted in Brazil for three consecutive years, the prevalence of *O. ovis* was 50% with the occurrence of the parasite in all seasons. Nevertheless, the highest prevalence (61.1%) were observed in the spring and summer. The climatic conditions of this region during this period are optimal for fly activity, and consequently for a high rate of sheep infestation (6).

The oestrosis therapy is based on the use of antiparasitic drugs that target the larvae. Many systemic antiparasitic drugs may be used for the oestrosis treatment. The most widely used antiparasitics to control *O. ovis* are closantel and the macrocyclic lactones, such as orally or injectable ivermectin (7, 8). Sheep treated with closantel, injectable and oral ivermectin had 100, 100, and 98% of efficacy, respectively considering all instars (7). In a survey conducted by Oliveira et al. (8) the injectable doramectin was 100% effective against all larval stages. Nevertheless, the first instar larvae (L1) is considered less susceptible to these drugs in comparison with the second instar larvae (L2) and third instar larvae (L3) (9) because the L1 larvae feed less thus they are less prone to ingest the systemic parasiticides (10). Martínez-Valladares et al. (11) observed that the long-acting injectable moxidectin had 90.2% of efficacy against L1.

The intensification of sheep farming and emergence of drug-resistant parasites have brought new challenges regarding the prophylaxis and treatment of small ruminant parasitism. Frequent cases of anthelmintic resistance have been reported (12–14) as well as ectoparasites, such as *Dermatobia hominis* (15, 16), *Rhipicephalus microplus* (17) and *Cochliomyia hominivorax* (18), with resistance to the treatments with macrocyclic lactones. For this reason, the aim of this work was to verify whether closantel and ivermectin that have been used for decades are still effective in sheep naturally infested with *O. ovis*. Because animals rarely die due to oestrosis, the economic losses caused by the parasitism may be underestimated. Therefore, the present study aimed also to assess the prophylactic effects of closantel and ivermectin on the productive performance of lambs.

## Methods

### Statement of Ethics

The study was carried out according to the standards established by the local Ethics Committee on Animal Use (FMVZ/UNESP protocol number 0159/2019).

### Study Area

The experiment was conducted in the experimental area of the São Paulo State University (UNESP), Department of Biostatistics, Plant Biology, Parasitology and Zoology of the Bioscience Institute, Botucatu, SP, Brazil.

The lambs were kept in an area of 3.840 m^2^ divided in 5 paddocks, in rotational grazing on *Urochloa decumbens*, exposed to natural infestation with *O. ovis*, and infection with gastrointestinal nematodes. The area had been grazed previously by sheep with natural infestation by those parasites. The animals had free access to tap water and mineral salt (Supre Ovinos - Coopermota^®^) and were fed daily with supplement with 16.5% of crude protein (Ração Ovinos Confinamento - Coopermota^®^) with an amount corresponding to 2% of their body weight.

### Experimental Design

The experiment was conducted from December 2019 to March 2020, during the rainy season. Thirty 2 to 3-month-old uncastrated male lambs crossbred Santa Ines x White Dorper were purchased from a local commercial farm. The lambs arrived in the experimental area on October 29th, and underwent an adaptation period of 37 days, before the beginning of trial on December 5th. Upon the arrival, blood and fecal samples from all the animals were collected for hematological and parasitological procedures, and in addition, all lambs were vaccinated against clostridiosis (Sintoxan Polivalente^®^ - Merial), and were treated with Toltrazuril (Farmacox^®^ - Farmabase, 20 mg/kg, orally) in order to prevent coccidiosis.

The animals were randomly distributed into three groups that were as homogenous as possible regarding body weight: group 1 (*n* = 10), control animals that did not receive any antiparasitic treatment against oestrosis; group 2 (*n* = 10), animals treated with ivermectin (Ivomec^®^ - Merial), 0.2 mg/kg, subcutaneous injection; and group 3 (*n* = 10), animals treated with closantel (Diantel^®^ - Merial), 10 mg/kg, orally. Treatments against oestrosis with closantel or ivermectin were performed on 5th December 2019 and on 13th February 2020.

Additionally, all experimental animals were treated with an anthelmintic combination with monepantel (2.5 mg/kg, Zolvix^®^ - Novartis), albendazole (10 mg/kg Endazol^®^ - Hipra) and levamisole phosphate (9.4 mg/kg Ripercol – Zoetis) for three consecutive days, which were administered in three different occasions: on 12th December, 16th January and 13th February. These anthelminitic treatments were performed to prevent losses due to nematode infections, keeping helminth infection degree similar among groups.

### Animals Performance and Clinical Observation

**Figure 1:**
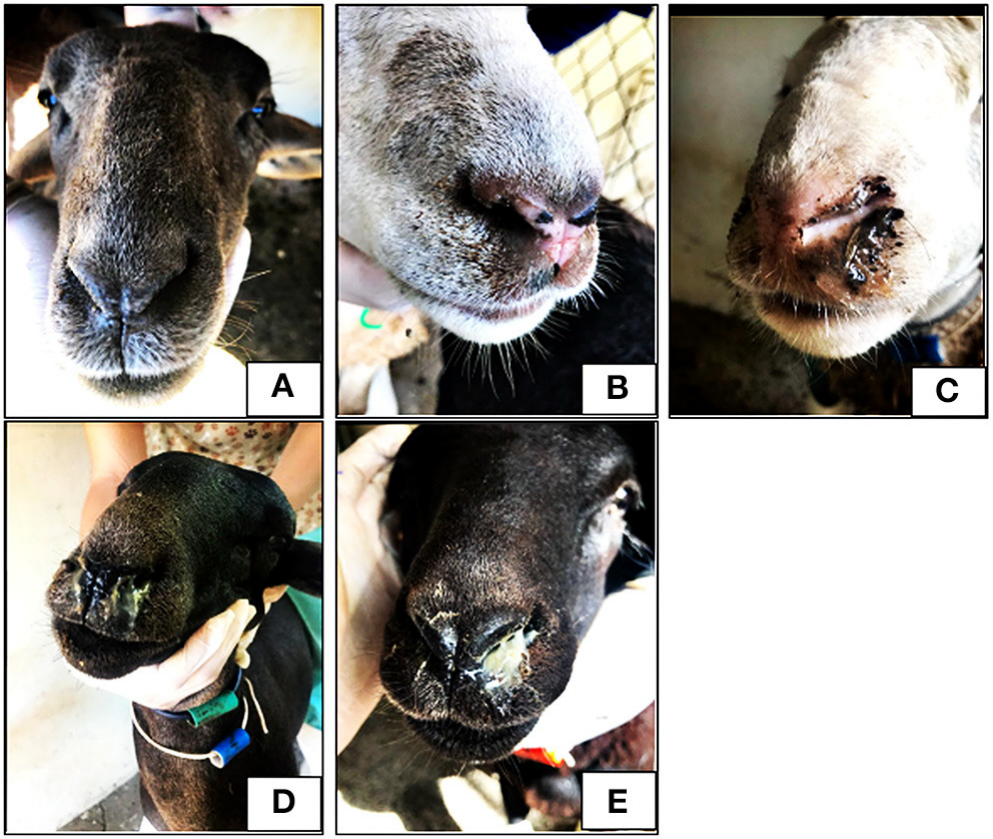
Photos of lambs with different nasal discharge score. No discharge **(A)**, serous discharge **(B)**, sero-mucous discharge **(C)**, thick mucous discharge **(D)** and mucopurulent thick discharge **(E)**.

Weekly, the lambs were weighed and examined looking for clinical signs of infestation by *O. ovis*, which included their nasal discharge degree (Figure 1; Table 1), recorded according to Dorchies et al. (7).

**Table 1:** Nasal discharge score.

**Score**	**Nasal discharge**
1 – Figure 1A	No discharge
2 – Figure 1B	Serous discharge
3 – Figure 1C	Sero-mucous discharge
4 – Figure 1D	Thick mucous discharge
5 – Figure 1E	Mucopurulent thick discharge

### Parasitological Analysis

Feces samples were obtained directly from the rectum of each animal weekly to perform fecal egg counts by modified McMaster technique, in which each worm egg counted represented 100 eggs per gram (EPG) (19). In addition, fecal cultures were performed for each group to obtain infective larvae (L3) of gastrointestinal nematodes, which were identified according to descriptions of Ueno and Gonçalves (19).

### Recovery of *Oestrus ovis* Larvae

**Figure 2:**
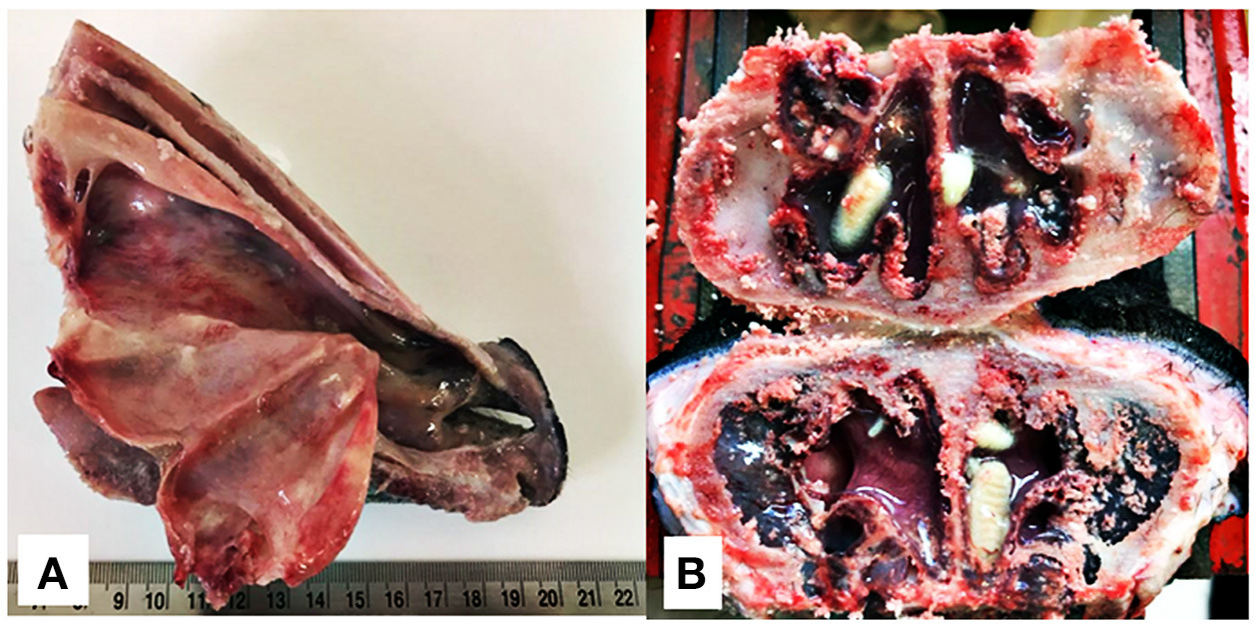
Longitudinal section of the rostral portion of the head without the mandible **(A)** and cross section of the nasal sinuses for the recovery of *O. ovis* larvae from naturally infested lambs **(B)**.

The animals were slaughtered 35 days after the last oestrosis treatment. They had their heads removed and then sectioned along their longitudinal and sagittal axis. Nasal cavity (nasal passage, septum, middle meatus, and conchae) (Figure 2A) and frontal sinus (Figure 2B) were carefully examined, and all larvae found were collected, counted, and identified with regards to their stage of development (1, 20).

### Recovery of Gastrointestinal Nematodes

One lamb from each group, with the highest EPG in the last sampling, was chosen for worm counts. After slaughter, the lambs had their abomasum and small intestine removed and opened longitudinally for content recovery. Their gastrointestinal contents were placed in graduated buckets to obtain aliquots of 10% of the total contents from each organ. The aliquots of 10% were stored in identified plastic flasks and preserved with 5% formaldehyde. All nematodes present in the 10% preserved material were morphologically identified and quantified according to their developmental stages (19).

### Hematological Analyses

Blood samples (5 ml) were obtained every 14 days by jugular vein puncture into Vacutainer^®^ tubes containing anticoagulant (K2 EDTA 7.2 mg, BD, Brazil). Packed cell volume (PCV) was determined by microhematocrit centrifugation (5 min/15,000 g, Fanem 211 microhematocrit centrifuge), and Total Protein Plasmatic (TPP) concentrations were estimated using a handheld refractometer (Refractometer SPR-N, Atago) (21, 22). Eosinophil counts were performed in a Neubauer's chamber after staining with Carpentier's solution, and the counts were expressed as the number of eosinophil cells per μL of blood (23).

### Enzyme-Linked Immunosorbent Assay

Plasma samples collected at 8 time-points were used to determine IgG levels against L2-soluble extract of *O. ovis*. The L2 extract was prepared as described by Silva et al. (24). A previously described protocol was applied to determine the parasite-specific plasma IgG levels (24) with some modifications: the plates were coated with 2 μg of antigen/mL, each wash was done six times which were incubated with peroxidase-conjugated rabbit anti-sheep IgG diluted at 1:5,000 then the plates were read at 450 nm by using an automated ELISA reader (Biotrak II; Amersham Biosciences, Little Chalfont, UK). For the negative control, plasma samples were obtained from young animals that were kept indoors without any contact with adult bot flies as previously described by Silva et al. (24). The plasma positive control sample used was from a sheep naturally infested with *Oestrus ovis*. Results were expressed as the percentage of plasma sample optical density (OD) value divided by OD of positive control.

### Therapeutic Efficacy

The therapeutic efficacy of each antiparasitic was based on the mean number of *O. ovis* larvae recovered from lambs of each group at necropsy and calculated according to the following Abbot's formula (25).


$$\begin{array}{l} \text{Efficacy}=\left(1-\text{Treated group}/\text{Control group}\right)\;\times \;100 \end{array}$$

For a therapeutic claim a reduction of larval counts should be at least 90% (25).

### Meteorological Data

The meteorological data was registered daily (temperatures, precipitation and relative humidity). The climatic conditions during the experiment period were similar to those reported in the last years, except for February with total rainfall of 552.2 mm. This high value occurred because on the 9th it rained 299 mm, more than expected for the whole month. Data was obtained from the Lageado Experimental Farm weather station belonging to Unesp - Campus Botucatu at a distance of 8 km from the experimental area.

### Statistical Analyses

All data were submitted to the normality test Shapiro-Wilk and transformed using log10 (x + 1) when necessary, which was the case of EPG, *O. ovis* larvae stages, eosinophils and IgG values. Data were analyzed by one-way analysis of variance (ANOVA) in the case of variables measured only once (carcass weight, daily BWG and larvae stages of *O. ovis*) and by ANOVA with repeated measures in the case of variables measured at several time points (body weight, PCV, TPP, OD and eosinophils) using the General Linear Model (GLM) of the Statistical Analysis System, version 9.2 (SAS Institute, Inc., Cary, NC, USA). The group means were compared by Tukey's test and chi-square test was used for categorical variable (nasal discharge). Values of *p* < 0.05 were considered statistically significant.

Descriptive statistical analyses were used to summarize the data on larval burden, in agreement with Bush et al. (26), using the following terms:

Prevalence: the number of hosts infested with *O. ovis* larvae, divided by the number of hosts examined;

Intensity of infestation: the number of *O. ovis* larvae in a single infested host;

Mean intensity of infestation: the total number of *O. ovis* larvae found divided by the number of hosts infected with that parasite.

## Results

During the study none of sheep showed adverse effects as a consequence of the treatments. Seven animals of the control group were infested with *O. ovis* larvae, which were alive. The control group average, considering also three animals with zero value, was 8.1 larvae per sheep (Table 2), which were 1.4 L1, 4.3 L2 e 3.0 L3 per head. An animal from control group with the highest intensity of infestation had 17 larvae (four L2 and 13 L3).

**Table 2:** First (L1), second (L2) and third stage (L3) larvae of *Oestrus ovis* in naturally infested lambs of the control, ivermectin and closantel groups.

**Group**	**Infested animal**	**Mean**	**Min–max**	**Total**		
				**L1**	**L2**	**L3**
Control (*n* = 10)	7	8.1^b^	0–17	14 (17%)	41 (51%)	26 (32%)
Ivermectin (*n =* 10)	3	0.6^a^	0–3	0	2 (33%)	4 (67%)
Closantel (*n* = 10)	0	0^a^	0	0	0	0
*p*-value		<0.001				

*Different letters in the column indicate a significant difference among means (Tukey test, p < 0.05)*.

The treatment with closantel was 100% effective against oestrosis, once there were no larvae on animals' heads. The treatment with ivermectin was 93% effective with three lambs presenting two larvae each in total six *O. ovis* larvae (Table 2). However, the larvae found were dead and had altered morphology, suggestive of the beginning of larva degeneration process. Differently from the lambs treated with ivermectin, the larvae identified in animals from the control group were active and without any morphological changes. Therefore, considering only alive larvae counting, the efficacy of ivermectin was 100%.

All the L1 observed in control group were in nasal conchae, 56% of the L2 were in ethmoidal conchae, and 44% in frontal sinus, while 23% of the L3 were in ethmoidal conchae and 77% in frontal sinus. Regarding the six dead larvae found in lambs treated with ivermectin, one L2 were in ethmoidal conchae, two L3 were in ethmoidal conchae and two L3 were in frontal sinus.

The clinical suggestive signs of oestrosis were scarce over the experimental period (Table 3) and there was no influence of treatment in the characterization of the lamb nasal discharge (*p* = 0.060). Each animal was examined 15 times over the trial. From the 450 nasal discharge exams conducted, 88% of them were serous, 9% were sero-mucous, 1% were very tick mucous, and 2% were mucopurulent thick (Table 3). This last score was observed in six animals, two from each group. Two weeks post the second oestrosis treatment, one animal from control group and one from group treated with closantel had muco-purulent discharges. Because lambs treated with closantel did not present larvae, it was possible to deduce that such discharge had no relation with *O. ovis* infestation.

**Table 3:** Total and percentage of nasal discharge classification of lambs naturally infested with *Oestrus ovis* of the control, ivermectin and closantel groups, in 15 evaluations from December 2019 to March 2020.

**Group**	**Serous**	**Sero-mucous**	**Thick mucous**	**Mucopurulent**	**Total**
Control	140 (94%)	8 (5%)	0	2 (1%)	150
Ivermectin	124 (83%)	19 (12%)	3 (2%)	4 (3%)	150
Closantel	133 (89%)	14 (9%)	0	3 (2%)	150
Total	397 (88%)	41 (9%)	3 (1%)	9 (2%)	450 (100%)

At the beginning of the trial, the average (±standard error) body weight were 23.1 (±1.3) kg, 22.8 (±1.4) kg and 22.9 (±1.6) kg, for non-treated control, ivermectin and closantel groups, respectively. In the last sampling, lambs were about 7–8 months old, and before slaughter, they had the following weight averages: 40.8 (±1.7), 40.5 (±2.0) kg and 40.6 (±1.6) kg for non-treated control, ivermectin and closantel groups, respectively. There was no significant difference among groups (*p* = 0.971) and also no significant group × time interaction (*p* = 0.615). The control group had daily gain mean (0.193 ± 0.016 kg), similar to ivermectin group (0.192 kg ± 0.01) and closantel group (0.192 kg ±0.02) (*p* = 0.994). In the same way, the means of carcass weight were similar (*p* = 0.999) among the different groups: 19.16 kg (±0.80), 19.11 kg (±1.01), 19.13 kg (±0.92), for control, ivermectin and closantel groups, respectively (Table 4).

**Table 4:** Initial and final body weight, daily body weight gain and carcass weight averages (±S.E.) of lambs naturally infested with *Oestrus ovis* of the control, ivermectin and closantel groups.

**Variable**	**Control**	**Ivermectin**	**Closantel**	***p*** **-value**
Initial	23.1 (±1.3)	22.8 (±1.4)	22.9 (±1.6)	0.971
Final	40.8 (±1.7)	40.5 (±2.0)	40.6 (±1.6)	0.990
Daily gain	0.193 (±0.02)	0.192 (±0.01)	0.192 (±0.02)	0.994
Carcass	19.16 (±0.80)	19.11 (±1.01)	19.13 (±0.92)	0.999

Because the animals had been kept in feedlot on their commercial farm of origin, EPG values were low and relatively homogeneous with averages similar between groups (*p* = 0.611) at the arrival in the experimental facilities. Lambs from control group had 70 (±39.58) EPG, lambs from ivermectin group had 740 (±322.90) EPG, and lambs from closantel group had 370 (±247.68) EPG.

**Figure 3:**
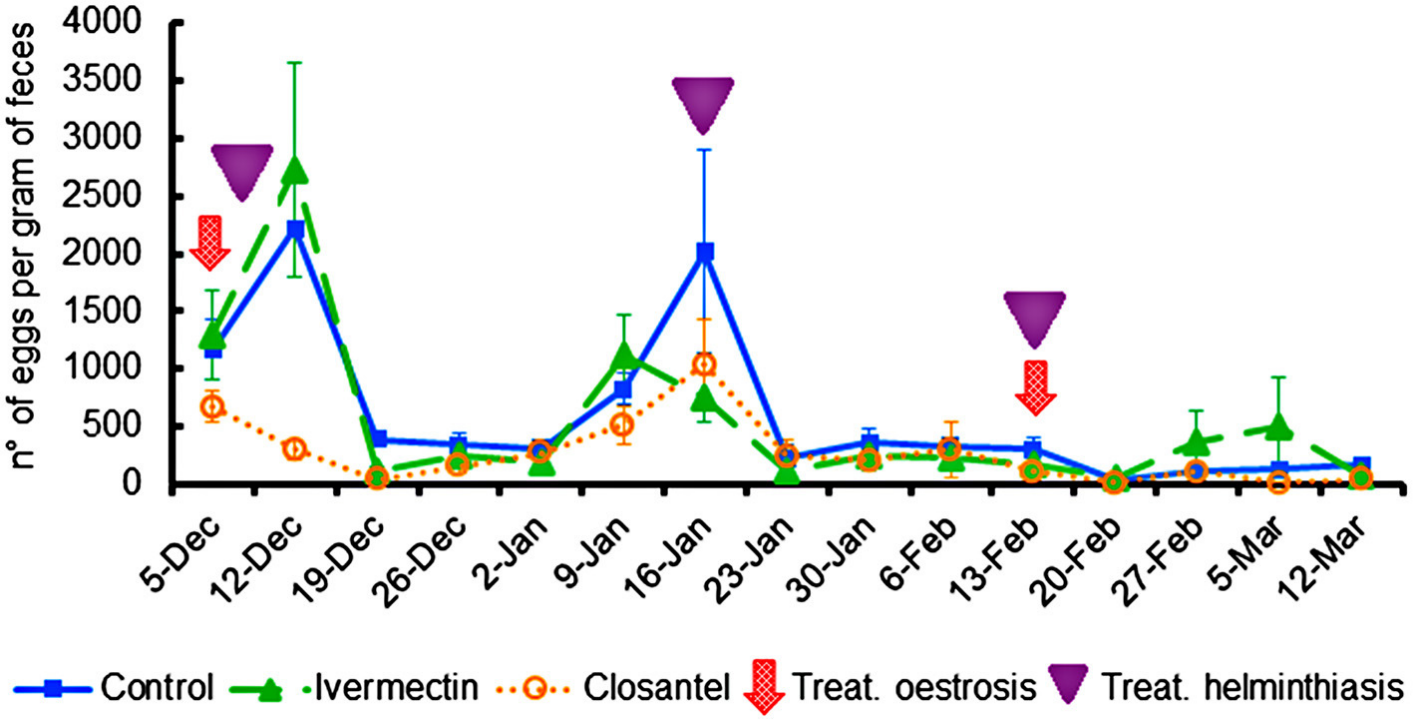
Averages of eggs per gram feces (±standard error) of lambs naturally infected with gastrointestinal nematodes and infested with *O. ovis*. The moments of treatments against oestrosis (with ivermectin or closantel) are indicated by arrow and treatments against helminthiasis (with a combination of levamisole + albendazole + monepantel) are indicated by triangle.

After the first treatment for oestrosis, closantel group had significantly lower (*p* < 0.001) EPG mean (300 ± 84) than the control (2,220 ± 424) and ivermectin (2,730 ± 925) groups. In order to avoid interference of helminthiasis in the animals' performance, it was chosen to drench all the animals with the combination of three anthelmintics (levamisole + albendazole + monepantel) on three occasions as previously described. By adopting this procedure, the EPG counts remained similar in all groups until the day of slaughter (Figure 3). Despite the low EPG values, group mean differences were observed on 19th December 2019, when the EPG average of the control group (390 ± 55) was higher (*p* < 0.001) than the means of the ivermectin (110 ± 48) and closantel (40 ± 22) groups; and on the last evaluation (12th March 2020), when once again the control group presented EPG mean (170 ± 49) significantly higher (*p* = 0.024) than the closantel (40 ± 40) and ivermectin (70 ± 30) groups. There was no significant interaction between the group and time (*p* = 0.281), but there was significant effect of time (*p* < 0.001) in the EPG counts.

It was also observed eggs of *Strongyloides* spp. and *Moniezia* spp. and oocysts of *Eimeria* spp. in fecal exams, however in small amounts. None of lambs had symptoms of parasitic gastroenteritis during the trial.

In fecal cultures, larvae of *Haemonchus* spp., *Cooperia* spp., and *Trichostrongylus* spp. were detected. However, *Haemonchus* spp. predominated during the experiment, being the only genus recorded from February until the end of experiment period (Table 5).

**Table 5:** Percentage of infective larvae of *Haemonchus* spp. (*Haem*)*, Trichostrongylus* spp. (*Trich*) e *Cooperia* spp. (*Coop*) in fecal cultures from control, ivermectin and closantel groups.

**Date**	**Control**			**Ivermectin**			**Closantel**		
	***Haem***	***Trich***	***Coop***	***Haem***	***Trich***	***Coop***	***Haem***	***Trich***	***Coop***
5/Dec	99	1	0	100	0	0	98	1	1
12/Dec	95	1	4	100	0	0	100	0	0
19/Dec	91	0	9	97	0	3	100	0	0
26/Dec	92	0	8	96	0	4	96	0	4
2/Jan	91	0	9	98	0	2	99	0	1
9/Jan	84	0	16	93	0	7	97	0	3
16/Jan	90	0	10	96	1	3	94	1	5
23/Jan	100	0	0	100	0	0	100	0	0
30/Jan	100	0	0	99	0	1	100	0	0
6/Feb	100	0	0	100	0	0	100	0	0
13/Feb	100	0	0	100	0	0	100	0	0
20/Feb	100	0	0	100	0	0	100	0	0
27/Feb	100	0	0	100	0	0	100	0	0
5/Mar	100	0	0	100	0	0	100	0	0
20/Mar	100	0	0	100	0	0	100	0	0

In the examination of the gastrointestinal contents, *Haemonchus* spp. was the major genus found, with a high number of immatures stages (Table 6). Additionally, ten adult females of *Trichostrongylus* spp. were recovered from a lamb treated with ivermectin and ten in an animal of the control group (Table 6).

**Table 6:** Total *Haemonchus contortus* and *Trichostrongylus* spp. worm burden from one lamb of each group naturally infected with gastrointestinal nematodes.

**Genera**	**Stage**	**Control**	**Ivermectin**	**Closantel**
*Haemonchus* spp.	Early L4	2,360	1,000	1,620
	Female late L4	2,540	10	180
	Male late L4	2,630	330	0
	Female early L5	370	0	0
	Male early L5	910	0	0
	Adult female	20	100	0
	Adult male	0	50	0
*Trichostrongylus* spp.	Adult female	10	10	0
	Total worm burden	8,840	1,500	1,800

**Figure 4:**
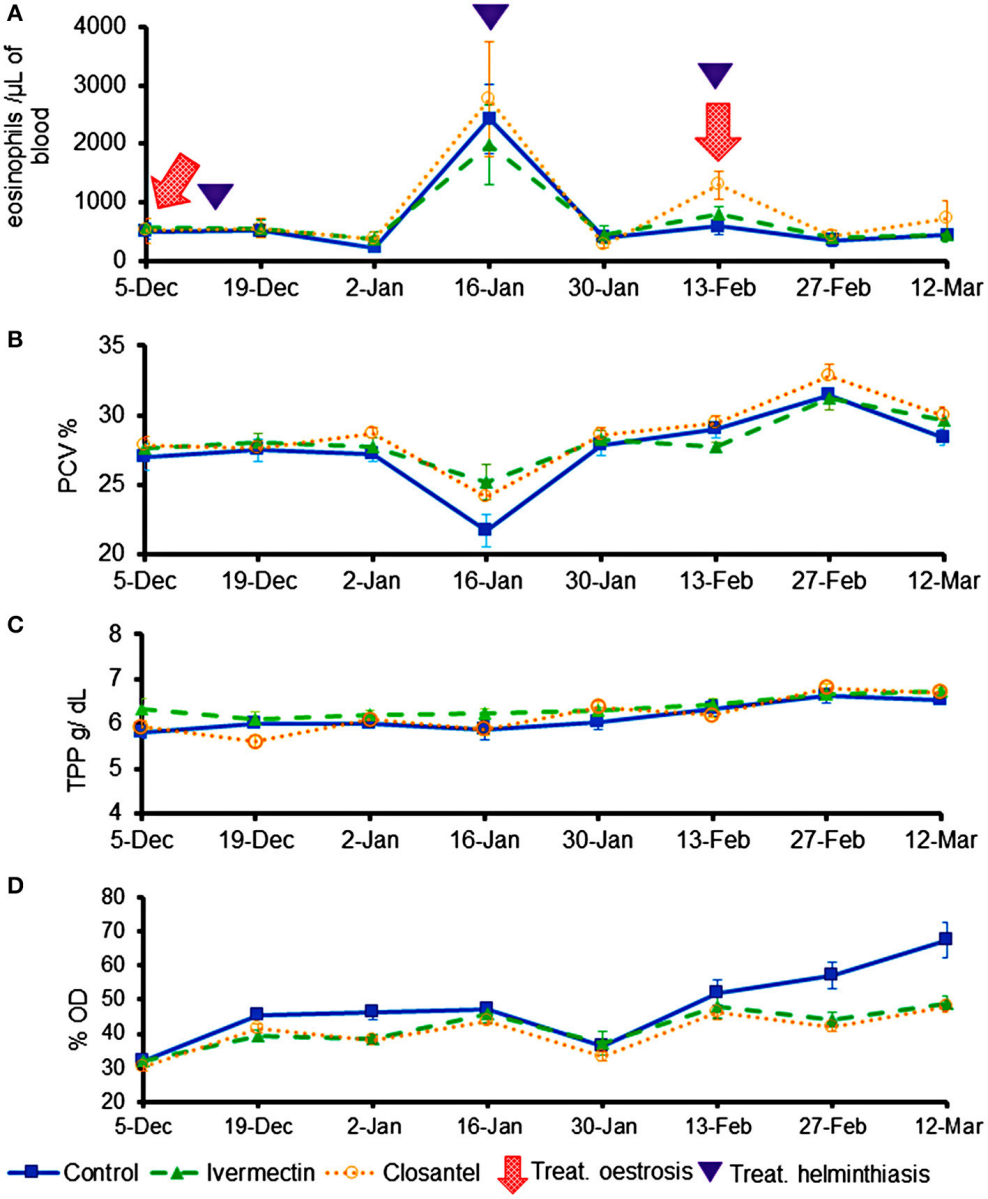
Averages (±standard error) of eosinophils (cells/μL) **(A)**, packed cell volume (%) **(B)**, total plasma protein (g/dL) **(C)**, and optical density (%OD) of IgG anti-*O. ovis* L2 **(D)** of lambs treated with ivermectin or closantel. The moments of treatments against oestrosis (with ivermectin or closantel) are indicated by arrow and treatments against helminthiasis (with a combination of levamisole + albendazole + monepantel) are indicated by triangle.

The values of eosinophils, PVC and TPP (Figure 4) were similar among the three groups throughout the trial, and there were no significant interactions between group and time for these variables. The only exception occurred on 19th December 2019, at the beginning of the experiment, when the TTP mean of ivermectin group (6.02 ± 0.109) was significantly higher than the closantel group (5.60 ± 0.126) (*p* = 0.017). The lowest means of PCV (Figure 4B) were registered on 16th January 2020 (*p* = 0.097), that were 21.7% (±3.65), 25.2% (±4.10) and 24.2% (±2.86), for the control, ivermectin and closantel groups, respectively. This reduction in the values of PCV coincided with the increase in the EPG means (Figure 3). Additionally, in the same date, it was observed the highest means of blood eosinophils (Figure 4A).

The mean levels of anti-*O. ovis* L2 IgG in plasma showed changes over time, resulting in significant interaction between time and treatment (*p* = 0.007). The antibody levels increased in the control group on the last 2 weeks of the trial, being significantly higher than values of the treated groups (*p* = 0.004) on 27th February and 12th March (Figure 4D). The three animals from control group that did not have any larvae of *O. ovis* presented the lowest averages of antibodies in their group (lamb 1: 49%; lamb 2: 49%; and lamb 4: 45%).

## Discussion

The results of this trial confirmed the high effectiveness of closantel in animals naturally infested by *O. ovis*, with the absence of larvae 35 days after the last treatment. Regarding the ivermectin, some treated animals still hosted larvae of second and third stages. Nevertheless, the larvae recovered from these animals were already dead presumably because of ivermectin action. However, it was not possible to determine when the larvae died. It is possible that the larvae have died soon after the treatment, but, somehow, they remained in the nasal cavities. The dead larvae are possibly expelled by sneezes that combined with the force of gravity cause nasal discharge.

Therefore, the results of the present study were in accordance with Dorchies et al. (7) that reported 100% of efficacy of oral closantel and injectable ivermectin against oestrosis, in sheep treated twice with an interval of 60 days. Likewise, oral ivermectin resulted in 100% efficacy against all stages 12 days after the treatment (9). Different of other Diptera that cause myiasis (*D. hominis* and *C. hominivorax*) with cases of drug-resistance (15-16-18), to the best of our knowledge, there is no report of populations of *O. ovis* with resistance to ivermectin or closantel.

The typical clinical signs of oestrosis were uncommon over trial and during the examination performed at necropsy there were no macroscopic morphological changes in the nasal cavities. There was only a small amount of secretion at the site where parasites were located and around the larvae. The absence of clinical sings in most of the evaluations may be a consequence of the low infestation rate. *Oestrus ovis* clinical manifestation is induced partially by spines on the larval cuticle and oral hooks used by L1 to move and progress toward nasal cavities and to anchor to the mucous surface. In addition, the L3 has large hooks, stout spines and dorsal plates which serve to support its gradual descend in the nasal cavities to the outside environment (4), whereupon this mechanical action provokes irritation in nasal mucosa (27). The pathogenesis of *O. ovis* is also induced by biomolecules (enzymes and antigens) excreted or secreted by the larvae onto the mucosa. These biomolecules comprise a complex array of enzymes necessary to degrade mucus and plasma proteins for larval feeding and nutrition (28). In agreement with our results, Silva et al. (6) observed that most tracer lambs in their study did not show any clinical signs typical of oestrosis after exposed to infestation during 4 weeks, although they hosted among 12 and 66 larvae, mainly L1 and L2. Like in the trial conducted by Silva et al. (6), possibly in our trial the infestation was recent, without enough time to manifestation of the immunopathological changes typical of the disease. Although not all infested sheep show clinical signs of oestrosis, the major symptoms of infestation (nasal discharge and frequent sneezing) are immune-mediated depending on acquisition of an immune response against the parasite (6, 29). Infestation induces a great recruitment of inflammatory cells such as mast cells and eosinophils, to site of host-parasite interaction and also increases the immunoglobulin production (29).

If the trial had a longer duration, perhaps the typical clinical signs would occur, because the ability of sheep to become resistant to oestrosis is limited, different from what usually happens in infection by helminths (24). Also drew attention the fact that the treatment against oestrosis did not influence the weight gain of the animals, demonstrating the benign character of the disease in lambs. The treatment with anthelminthics, including in those lambs from control group, avoided the interference of helminthiasis in the body weight gain of the lambs, which were around 190 g per day, within the expected for grass fed male lambs supplemented with concentrate (30). However, more studies are necessary to evaluate the influence of oestrosis on sheep productivity in our environmental conditions, especially in older animals continuously exposed to the parasitism.

Lopes et al. (31) reported a case of poisoning by closantel in three animals from a group of 15 sheep treated with closantel twice with interval of 28 days. These lambs showed apathy, anorexia, diarrhea and blindness after the second treatment with 7.5 mg/kg orally. In our trial, lambs did not show any adverse effects post treatment with closantel or ivermectin. Some studies have shown that well-nourished animal, which was the case of our lambs, has less chances of poisoning because the closantel binds to plasmatic proteins, mainly albumin and healthy animals tend to have higher plasmatic protein level that reduce the amount of drug available in tissues (32, 33).

There was an increase in EPG means and a reduction in PCV values caused by *Haemonchus* spp. in all groups on 16th January 2020. After the anthelmintic treatment, PCV means increased and remained within the reference interval for sheep until the end of the study. Concomitantly, it was observed a significant increase in blood eosinophils counts on 16th January. Eosinophilia has been reported as one of the defense mechanisms against the gastrointestinal nematode parasitism (34, 35) and against *O. ovis* infestation (36).

While IgG-anti *O. ovis* means remained low in treated groups, the control lambs showed an increase in averages at the end of the trial, thus indicating that the animals were responding to parasite infestation. Previous studies demonstrated that detection of *O. ovis* antibodies in sheep is a valid diagnosis technique (24, 37–39).

## Conclusions

Closantel oral drench at a dose rate 10 mg/kg and ivermectin subcutaneously at 0.2 mg/kg remain effective in the prophylaxis of oestrosis. These drugs are effective at the same concentrations used in the previous studies and there is no evidence of increasing trends of resistance. At the early stage of infestation, *O. ovis* parasitism did not cause a negative impact on the performance of lambs.

## Data Availability Statement

The original contributions presented in the study are included in the article/supplementary material, further inquiries can be directed to the corresponding author/s.

## Ethics Statement

The animal study was reviewed and approved by Ethics Committee on Animal Use (FMVZ/UNESP Protocol Number 0159/2019).

## Author Contributions

HB performed all experiment, collected and analyzed the data, and completed the manuscript preparation. JL, ACA, and GF helped in the collection of data. AFA, JL, ACA, and MA participated in its design and coordination and helped to interpret the results. All authors interpreted the results and substantively revised the manuscript, read, and approved the final manuscript.

## Funding

HB and JL received scholarship from Coordenação de Aperfeiçoamento de Pessoal de Nível Superior (CAPES), ACA was in receipt of a fellowship from Fundação de Amparo à Pesquisa do Estado de São Paulo (FAPESP) (grant #2021/03479-1), GF received scholarship from PIBIC-CNPq, and AFA was in receipt of a fellowship from Conselho Nacional de Desenvolvimento Científico e Tecnológico (CNPq) (grant #305187/2017-1). This study was funded by Fundação de Amparo à Pesquisa do Estado de São Paulo (FAPESP) (grant #2019/25185-0).

## Conflict of Interest

The authors declare that the research was conducted in the absence of any commercial or financial relationships that could be construed as a potential conflict of interest.

## Publisher's Note

All claims expressed in this article are solely those of the authors and do not necessarily represent those of their affiliated organizations, or those of the publisher, the editors and the reviewers. Any product that may be evaluated in this article, or claim that may be made by its manufacturer, is not guaranteed or endorsed by the publisher.
